# Religious Coping in Parents of Children with Down Syndrome: A Systematic Review of the Literature

**DOI:** 10.1007/s10943-024-02207-0

**Published:** 2025-01-23

**Authors:** Hüseyin Çaksen

**Affiliations:** Divisions of Pediatric Neurology and Genetics, and Developmental-Behavioral Pediatrics Department of Pediatrics, Faculty of Medicine, Necmettin Erbakan University, 42090 Meram, Konya, Türkiye

**Keywords:** Religion, Spirituality, Religious coping, Parent, Children, Down syndrome

## Abstract

Having a child with Down syndrome (DS) is stressful for families. Social, physical, economic and emotional difficulties are the most challenging stressors for parents of children with DS. Therefore, parents who have children with DS have used various types of coping strategies. This systematic review investigates religious coping in parents of children with DS to synthesize what is known of the literature regarding religious coping among parents of children with DS. Pubmed and Scopus databases were searched with no restriction on language and the year of publication. Additionally, manual searches of the retrieved articles’ references were performed. Forty-four original studies published between 2000 and 2023 were included in the review. No study on the subject was found in either Pubmed or Scopus before 2000. Most studies were conducted in USA 7 (15.9%), Egypt 7 (15.9%), and Türkiye 6 (13.6%). In the studies, the total number of participants was 4266, range between 8 and 530 (median 55.5). The ethnic origins of the participants were very diverse and the studies included participants from many cultures around the world. Based on the studies, we identified seven themes that represented the main stressors encountered by parents/caregivers of children with DS: “information deficits,” “child caregiving burdens,” “familial difficulties,” “financial difficulties,” “challenges related to social and professional support,” “society’s misconceptions,” and “worries about the future.” The most commonly (*n* = 12, [27.2%]) used coping scale was coping orientation to problems experienced. Religious coping was the coping strategy most frequently used by participants in 36 (81.8%) studies. Religion, spirituality, and belief in Allah (God) were of central importance for most participants in most of the studies. Most parents reported that belief in Allah (God) encouraged them to accept the diagnosis of DS and feel better and become stronger; provided improvements in the lives of families and the necessary resources to face their difficulties; played a fundamental role in adaptation with the conditions of their children with DS; brought them peace of mind and a sense of hope; and motivated them to keep on moving forward. In conclusion, religion plays an important role in the lives of most parents of children with DS. Religious coping has been used by parents of children with DS in many cultures around the world, regardless of religion, race, or ethnicity. Belief in Allah (God), belief in fate and belief in the afterlife, provided physical, mental and psychosocial relief for most parents of children with DS.

## Introduction

Down syndrome (DS) is the most common chromosomal condition associated with intellectual disability and is characterized by a variety of additional clinical findings (Bull, 2020). This syndrome occurs when there is an extra copy of the 21st chromosome (trisomy 21). It occurs in approximately 1 of 800 births worldwide (Bull, 2020). The health issues and life trajectory of persons with DS are complex, and the condition is associated with many disparate medical, psychological, and social issues from infancy through adulthood (Bull, 2020).

Having a child with DS is stressful for families. Social (social contacts, keeping a secret, social reactions, family relations), physical, economic and emotional difficulties are the most challenging stressors for parents of children with DS (Sari et al., 2006). Parents who have children with DS have used various types of coping strategies including “attribution techniques in accepting conditions that occur at the moment,” “asking for a positive attitude to be more religious and get closer to God” and “choosing a lot of outdoor activities with children as an effort to get out” (Nurmalita & Kristiana, 2019).

Religion is a declaration, a manifesto, describing both the One who made this beautiful universe and the universe itself (Nursi, 2012). Religion is a law that Allah communicated to smart people through the prophets that leads people to peace, goodness, blessings and salvation in this world and in the hereafter (Tümer, 1994). Spirituality is the high states, blessings, tastes, and happiness that a person feels in his own conscience and soul when he fulfills the de facto gratitude by acting in accordance with Allah's orders and prohibitions with his material organs, intangible feelings, and letaif (the plural form of latife; subtle faculty) (Çaksen et al., 2024).

Religion is broader than spirituality and it encompasses spirituality. Spirituality is a dimension of religion. So, religion and spirituality are not the same things (Çaksen et al., 2024). However, in the literature, the terms of spiritual and spiritual coping are sometimes used instead of or synonymously with religious and religious coping, respectively (Gallagher et al., 2015).

Coping refers to the intentional efforts we engage in to minimize the physical, psychological, or social harm of an event or situation (Carroll, 2013). Religious coping is a religiously framed cognitive, emotional, or behavioral response to stress that encompasses multiple modalities and goals, as well as positive and negative dimensions. Gaining meaning in life can serve many purposes, including closeness to God, hope, peace, connection with others, personal growth, and personal restraint (Wortmann, 2013).

One might turn to religion when under stress for a widely variety of reasons: religion might serve as a source of emotional support, as a vehicle for positive reinterpretation and growth, or as a tactic of active coping with a stressor (Carver et al., 1989). Considerable literature suggests that some aspects of religion are consistently associated with adjustment to illness, and evidence for religion as a stress buffer and as a stress deterrent were found. Potential pathways by which religion may influence adjustment to illness were also delineated, including: providing an interpretive framework or cognitive schema; enhancing coping resources; and facilitating access to social support and promoting social integration (Siegel et al., 2001).

This systematic review investigates religious coping in parents of children with DS. To the best of our knowledge, this systematic review is the first to examine religious coping among parents of children with DS in the literature. The aim of this review is to synthesize what is known of the literature regarding religious coping among parents of children with DS.

## Methods

This systematic review was conducted following the Preferred Reporting Item for Systematic Reviews and Meta-Analysis (PRISMA) 2020 statement, an updated guideline for reporting systematic reviews (Page et al., 2021).

### Eligibility Criteria

Studies were included if they evaluated religious coping in parents/caregivers of children with DS. The review included parents/caregivers of all ages and from all settings. We included all articles on the subject published in English or other languages. Questionnaire-based studies, qualitative interviewing studies, observational qualitative studies and quantitative studies were included.

Studies involving parents of children diagnosed prenatally with DS and non-religious coping were not included. Non-original research articles such as reviews, case reports including a few cases (or a family), editorials, letters, notes, book reviews, etcetera were excluded from the review. Dissertations and theses were also not included in the review.

### Search Strategy

A systematic literature search was performed to identify published studies investigating religious coping in parents of children with DS. Pubmed and Scopus databases were searched for eligible studies with no limit on publication year (i.e., from inception to the search date) on September 5, 2023. Additionally, manual searches of the retrieved articles’ references were performed. Medical Subject Heading terms and suitable keyword synonyms related to “religious” and “spirit” (including the related terms of religiousness, religiosity, religion, spiritual, and spirituality) in combination with “coping” (including the related term “cope”), “parent” “caregiver” and “Down syndrome” were used.

Two separate searches were performed in Pubmed using the related keywords in order not to miss the studies on the subject. The details of the search strings in Pubmed and Scopus were as follows: Pubmed 1: (((religious) OR (spiritual)) OR (coping)) AND (“Down syndrome”); Pubmed 2: (religi*[Title/Abstract]) OR (spirit*[Title/Abstract]) OR (coping[Title/Abstract]) OR (parent[Title/Abstract]) OR (caregiver[Title/Abstract]) AND (Down syndrome[Title/Abstract]); and Scopus: ( TITLE-ABS-KEY ( religi*) OR TITLE-ABS-KEY ( spirit*) OR TITLE-ABS-KEY ( coping) AND TITLE-ABS-KEY ( “Down syndrome”)). All search results were imported into Mendeley reference management software.

### Study Selection Process

The included studies were first evaluated by an initial review of the article title and an abstract review. Then, the full texts of the publications that met the inclusion criteria were taken for data extraction. The review was not registered and a protocol was not prepared. An investigator (HÇ) conducted the study selection process and review.

### Data Extraction

A standardized data extraction form was developed to obtain information relevant to this systematic review. The form included the authors’ information, publication year, type of study, sample size, country of study, ethnic background of participants, religion of participants, used scales to measure stress, anxiety, depression, coping (etc.,), significant stressors, and major findings of study.

### Data Synthesis

A narrative approach is recommended in systemic reviews to evaluate studies that vary in design (Popay et al., 2006). So, a qualitative narrative synthesis was used to summarize the findings of this review because to the methodological heterogeneity of the included studies.

## Results

### Study Selection

A total of 1516 records were identified: 1499 from the electronic bibliographic database searches and 17 from the search of the included articles’ reference lists. Of these, 344 duplicates were removed. After screening the titles and abstracts, only 70 studies potentially met the eligibility criteria for inclusion; full text versions of these studies were retrieved for review. Of these, 22 were excluded because they involved non-religious coping of parents of children with DS. Two studies were excluded due to few numbers of children with DS. One study was excluded because of review article including religious and non-religious coping of parents of children with DS. As a result, 44 studies were included in the present systematic review.

**Fig. 1:**
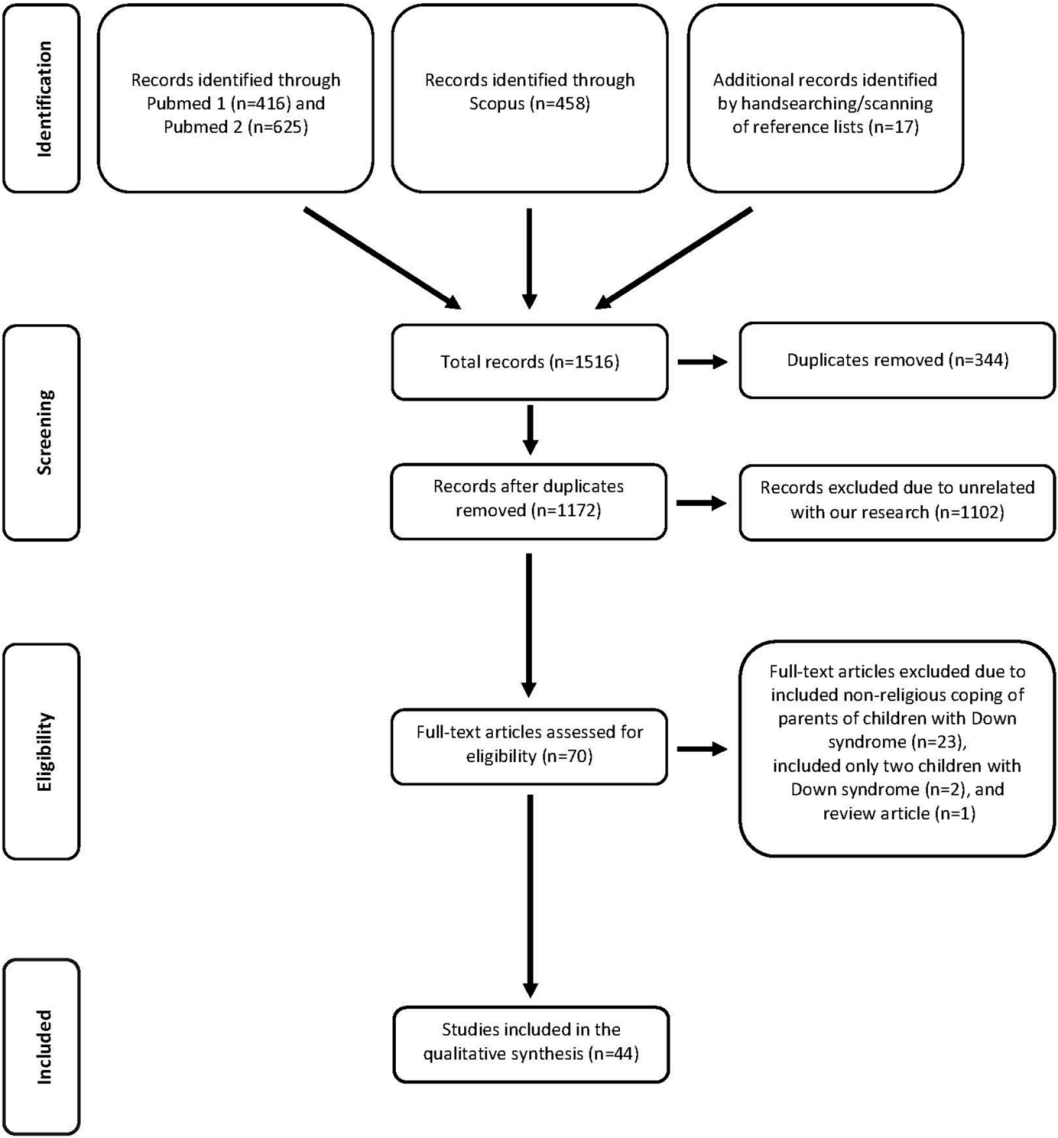
A PRISMA flowchart describing the search results and selection process

A PRISMA flowchart describing the search results and selection process is seen in Fig. 1. Although six articles (Boehm & Carter, 2019; Ezeonu et al., 2021; Gallagher et al., 2015; Gokgoz & Kabukcuoglu, 2022; Isa et al., 2017; Karaca & Konuk Şener, 2021) were indexed in Pubmed, they were not found in searches using either keyword groups. Four of them (Ezeonu et al., 2021; Gokgoz & Kabukcuoglu, 2022; Isa et al., 2017; Karaca & Konuk Şener, 2021) were obtained from Scopus, the other two studies (Boehm & Carter, 2019; Gallagher et al., 2015) were identified by handsearching/scanning of reference lists.

### Description of Included Studies

#### Designs and Countries of Studies

Studies about religious coping among parents/caregivers of children with DS are shown in Appendix 1. The 44 included studies were published between 2000 and 2023. No study on the subject was found in either Pubmed or Scopus before 2000. Thirty (68.1%) studies were performed after 2015. The most studies (*n* = 30, [68.1%]) (Al Rubaie, 2022; Amireh, 2019; Barakat & Mohamed, 2019; Barros et al., 2017; Behrani & Shah, 2016; Boehm & Carter, 2019; Celik & Kara Uzun, 2023; Cless et al., 2018; Darla & Bhat, 2021; Ezeonu et al., 2021; Fatima & Suhail, 2010; Gallagher et al., 2015; Gupta et al., 2013; Hafez et al., 2023; Hayat & Zafar, 2015; Herken et al., 2000; Isa et al., 2017; King, 2001; Lee et al., 2021; Mahmoud et al., 2022; Mohammed et al., 2020, 2021; Negm et al., 2022; Nelson Goff et al., 2013, 2016; Norizan & Shamsuddin, 2010; Pruktarat et al., 2021; Qahhar & Khan, 2019; Sullivan, 2002; van Riper, 2007) were questionnaire-based study. Seven (15.9%) studies (Alcantara & Castronuevo, 2016; Braga et al., 2021; Gashmard et al., 2020; Gokgoz & Kabukcuoglu, 2022; Gören, 2015; Lam & Mackenzie, 2002; Sheets et al., 2012) were a semi-structured interview. Six (13.6%) studies (Duarte et al., 2022; Huiracocha et al., 2017; Karaca & Konuk Şener, 2021; Özkan & Günaydın, 2022; Pillay et al., 2012; Suza et al., 2020) were observational qualitative studies, based on in-depth interviews. The remainder study by Elmwafie et al., (2022) was a quasi-experimental design.

The most studies were reported from USA 7 (15.9%), Egypt 7 (15.9%), and Türkiye 6 (13.6%). The remainders were as follows: India 3 (6.8%), Pakistan 3 (6.8%), Brazil 3 (6.8%), United Kingdom 2 (4.5%), Malaysia 2 (4.5%), China 1 (2.2%), Australia 1 (2.2%), Philippines 1 (2.2%), Ecuador 1 (2.2%), Jordan 1 (2.2%), Iran 1 (2.2%), Indonesia 1 (2.2%), Thailand 1 (2.2%), Canada 1 (2.2%), Nigeria 1 (2.2%), and Saudi Arabia 1 (2.2%). All of the studies were in English except two studies (Gören, 2015; Herken et al., 2000), in Turkish.

#### Participant Characteristics

In the studies, the total number of participants was 4266, varied between 8 and 530 (median 55.5). Of 44 studies, 17 (38.6%) included mothers only, and 27 (61.3%) included mothers and fathers with or without other family members or caregivers. Mothers and their children with DS were examined in two studies (Barros et al., 2017; Elmwafie et al., 2022). Eight studies (Amireh, 2019; Behrani & Shah, 2016; Boehm & Carter, 2019; Ezeonu et al., 2021; Gallagher et al., 2015; Isa et al., 2017; Karaca & Konuk Şener, 2021; King, 2001; Özkan & Günaydın, 2022) included both children with DS and patients with developmental disabilities, intellectual disability, learning disabilities, and/or autism.

The number of children with DS was not noted in three studies (Behrani & Shah, 2016; Karaca & Konuk Şener, 2021; King, 2001). The study by Nelson Goff et al., (2013) included parents of children with DS diagnosed during both the prenatal and postnatal periods, but prenatal results were not included in our study. Four studies (Barros et al., 2017; Fatima & Suhail, 2010; Herken et al., 2000; Nelson Goff et al., 2013) included a control group.

Ethnicity of the participants was recorded in all of the studies except for the study by Sullivan (2002). The ethnic origins of the participants were very diverse and the studies included participants from many cultures around the world (Appendix 1). The participants were Muslim in most studies (*n* = 21, [47.7%]), though the religion of the participants was not stated in seven (15.90%) of the studies. In the other studies, the participants' religions were Christian (*n* = 8, [18.1%]), participants from various religions (*n* = 6, [13.6%]), Buddhist (*n* = 1, [2.2%]), and Confucian (*n* = 1, [2.2%]).

#### Using of Coping Scales

A variety of coping scales were utilized in the studies. The most common (*n* = 12, [27.2%]) used coping scale was coping orientation to problems experienced. This brief coping orientation to problems experienced consists of 14 scales, of two items each: (1) active coping, (2) planning, (3) positive refraining, (4) acceptance, (5) humor, (6) religion, (7) using emotional support, (8) using instrumental support, (9) self-distraction, (10) denial, (11) venting, (12) substance use, (13) behavioral disengagement, and (14) self-blame (Carver, 1997).

The other used coping scales were various interview scales including quantitative measures and/or qualitative questions, breaking difficult news, qualitative design, Chinese parental coping scale, classic grounded theory approach, etcetera. Additionally, a variety of depression, anxiety, stress scales such as Beck depression inventory, perceived stress scale hospital anxiety depression scale, parental stress scale, etcetera were used in some studies. A detailed description of the scales that were used is presented in Appendix 1.

#### Stressors in Parents of Children with DS

Parents of children with DS have high level of stress (Barakat & Mohamed, 2019; Isa et al., 2017; Norizan & Shamsuddin, 2010). The depression score of parents of children with DS was higher than the control group. The mothers had higher depression scores than fathers (Herken et al., 2000). Parenting stress among mothers of children with DS was significantly correlated with presence of maternal depressive, anxiety and stress symptoms and it significantly differed by behavioral problems of their children (Norizan & Shamsuddin, 2010). However, Amireh (2019) noted that parents of typically developed children showed non-significantly higher levels of stress than the parents of DS children.

No specific stressors were identified in ten studies (Boehm & Carter, 2019; Cless et al., 2018; Fatima & Suhail, 2010; Gallagher et al., 2015; Gupta et al., 2013; Hayat & Zafar, 2015; Herken et al., 2000; Isa et al., 2017; Qahhar & Khan, 2019; Sullivan, 2002). After reviewing the reminder 34 studies, we identified seven themes that represented the main stressors encountered by parents/caregivers: “information deficits,” “child caregiving burdens,” “familial difficulties,” “financial difficulties,” “challenges related to social and professional support,” “society’s misconceptions,” and “worries about the future.” Financial difficulties were noted in 13 studies, nine from developing countries (Appendix 1). Table 1 shows main stressors and psychosocial benefits of belief in God for parents/caregivers of children with DS.

**Table 1:** Main stressors and psychosocial benefits of belief in God for parents/caregivers of children with Down syndrome

Main stressors	Psychosocial benefits of belief in God
Information deficits	Encouragement to accept the diagnosis of Down syndrome
Child caregiving burdens	Encouragement to feel better and to become stronger
Familial difficulties	Providing improvements in the lives of families
Financial difficulties	Providing the necessary resources to face parents' difficulties
Challenges related to social and professional support	Playing a fundamental role in adaptation with the condition of the child with Down syndrome
Society’s misconceptions	Bringing peace of mind and a sense of hope
Worries about the future	Motivation to keep on moving forward

#### Coping Strategies in Parents of Children with DS

The birth of an infant with DS was difficult and stressful experience for parents. Most parents used coping strategies mentioned by Lam and Mackenzie, (2002) and Braga et al., (2021). In the first days after birth, internal and external resources, which represent coping, are developed to accept and refocus ideas and feelings toward DS. Over time these resources have an effect and cause changes in family functioning patterns and in the relationship of these families with the outside world, as they seek support for child development (Braga et al., 2021).

The used coping strategies were self-reliance, seeking social support, and avoidance in the following months. Parents sent their children to an integrated or private childcare center about the age of 2 years. Both emotion-focused and problem-focused coping were employed in this stage. The types of stressors changed over time according to the child's age, and coping strategies varied accordingly (Braga et al., 2021; Lam & Mackenzie, 2002).

Religious coping, emotion-focused coping (avoidance and acceptance of child’s situation), and problem-focused coping (using emotional support and seeking social support) were frequently used coping styles in the studies. Religion, spirituality, and belief in God were of central importance for most participants in most of the studies. Most parents reported that belief in God encouraged them to accept the diagnosis of DS and feel better and become stronger; provided improvements in the lives of families and the necessary resources to face their difficulties; played a fundamental role in adaptation with the conditions of their children with DS; brought them peace of mind and a sense of hope; and motivated them to keep on moving forward (Table 1).

Some parents noted that God gave them His grace to deal with the medical, physical, cognitive and social challenges (Nelson Goff et al., 2013) and they simply could not have survived the rigors of their caregiving careers without God’s help (King, 2001). Some parents also believed the truth of “God does not burden any soul with more than it can bear (Boehm & Carter, 2019; King, 2001)” which is an ayat in the Quran (Surah Al-Baqarah, 2024). However, Huiracocha et al., (2017) noted that while religious beliefs served as an important resource, they render mothers vulnerable to the chastisements of priests for some mothers.

Contrary to the above studies, Fatima and Suhail (2010) reported that the more the mothers of children with DS believed in immanent justice, the more anxiety they experienced the previous week. The authors explained this unexpected result as follows: It is probable that by believing in immanent justice mothers of these children might have thought that their child’s diagnosis was some sort of punishment for them, increasing the psychological burden on mothers.

Gallagher et al., (2015) noted that parents of children with developmental disabilities including DS who reported higher spirituality exhibited more depressive symptomatology and were more likely to reach the threshold for possible caseness. Associations between spirituality and depression in these parents are more complex than previously thought. Spirituality was used most frequently when parents felt alone and unsupported. It appears to be an integral part of how parents caring for children with developmental disabilities respond to stressful situations woven into the very nature of their lives (Gallagher et al., 2015).

Religious coping was the most common coping strategy used by participants in 36 (81.8%) studies. Fatima and Suhail (2010) studied beliefs in immanent and ultimate justice, closely related to religious doctrines, in mothers of DS children, although they did not note any terms of religion, spirituality, or faith in Allah in their article. Religious teachings endorse notions of ultimate justice (a misfortune is compensated in the long run) and immanent justice (a misfortune is caused by previous misdeeds) (Harvey & Callan, 2014).

Among the remainder eight studies, Sullivan (2002) and Behrani and Shah (2016) noted that some participants turned to religion. Pruktarat et al., (2021) and Lee et al., (2021) recorded that some participants sought spiritual support. In the study of Nelson Goff et al., (2016) some parents described a stronger faith brought about from their spiritual growth and they frequently citied their religion or being spiritual as important. Belief in God was resource adopted for some participants in another study (Braga et al., 2021). Gupta et al., (2013) noted that probably, religious and spiritual beliefs also helped in coping strategies of parents. Lam and Mackenzie (2002) noted a term of “appeal to a supernatural power” among the Chinese parental coping scale used in the study; however, they did not record any information about this coping style in the findings and discussion section of the article.

## Discussion

Our systematic review showed that parents/caregivers of children with DS had high level of stress in the most studies, but Amireh (2019) reported that parents of DS children showed non-significantly lower levels of stress than the parents of typically developed children. Based on the most studies, we identified following themes about stressors: “child caregiving burdens,” “familial difficulties,” “financial difficulties,” “information deficits,” “society’s misconceptions,” “worries about the future,” and “challenges related to social and professional support.” The difficulty of the process has been reported by parents with disabled children under the following sub-themes in the literature: shock/collapse, not attributing the diagnosis, fear of losing the child, disappointment, self-blame, loneliness, lack of paternal support, negative environmental effects, concern for the future, and despair (Hatun et al., 2016).

Mothers of children with DS show higher levels of parental stress than mothers of typically developing children (Phillips et al., 2017). Both internalizing and externalizing problems correlated with maternal stress in mothers of children DS (Fucà et al., 2022). Parents of children with DS who quantitatively experienced high stress or low stress used different behavioral themes to describe their experience qualitatively. Positive appraisals, resources, and ability to engage in problem solving and coping were associated with family resiliency (Hall et al., 2012). In parents of children with DS, using acceptance, rumination, positive refocusing, refocusing on planning, and catastrophizing to a greater extent was related to more stress, whereas using positive reappraisal more often was related to less stress in parents of children with DS (van der Veek et al., 2009).

Parents of children with DS with high social support and resilience have lower level of parenting-stress. Resilience and social support-oriented therapy appears to have efficacy for helping parent-carers of children with DS to manage their stress arising from raising such children (Onyedibe et al., 2018). Gender of parents, family demands, and family appraisal were significantly associated with individual health. Age of the child with DS, family demands, and family appraisal significantly accounted for family functioning (Hsiao & Van Riper, 2011). Families having older children with DS, greater parental education, higher family income, fewer family demands and greater social supports contributed to healthier family functioning (Hsiao, 2014).

In this systemic review, we found that religious coping was the most commonly used coping strategy 36 (81.8%) used by participants in the studies. The other frequently used coping styles were avoidance, acceptance of child’s situation, using emotional support and seeking social support. The coping resources of the parents with disabled children were noted in the literature as follows: expert support, family support, environmental care, rationalizing, hope, following daily routines, blaming, and unable to be apart from the child (Hatun et al., 2016).

The religious coping resources were acceptance (accepting what comes from Allah and destiny), giving spiritual meaning (being tested, repentance, fear of Allah, and being an entrustment from Allah), and trust in Allah (resignation, gratitude, and prayer) (Hatun et al., 2016). The theme of prayer has been noted to be repeated often during the interviews with parents. It is understood from parents’ statements that parents who pray talk with Allah and reduce their loneliness, foster their hopes and reflect their trust in Allah through prayer, and strengthen themselves psychologically (Hatun et al., 2016). Our findings about the religious coping resources, obtained from the studies, were similar to these literature data.

Caregivers, who mostly comprised of mothers, struggled to accept the diagnosis initially that led them to search for answers to many of their concerns about raising a person with DS. For the caregivers, dealing with health conditions that persons with DS suffered from was initially difficult. Caring for these individuals led to heavy impact upon their caregivers’ own lives who took extraordinary efforts to cope with the burden. Seeking quality education for the persons with DS and participation in social activities was also challenging (AlShatti et al., 2021).

As a result of their experiences, families who had children with DS reported becoming more certain about what matters. Families adopted perspectives of optimism, acceptance, and appreciation, and of striving to change the environment or to meet their children’s needs as well as possible (King et al., 2009). The narratives of mothers of children with DS highlight the process of meaning-making that these mothers engaged in, their resistance to the dominant discourse on disability, and their eventual transformations in perceptions of disability and motherhood (Lalvani, 2008).

Parent coping variables were the strongest predictors of both positive gain and parental distress, with reframing emerging as a predictor of positive gain and parent empowerment emerging as a predictor of both greater positive gain and lower parental distress in parents of children with intellectual disabilities/developmental delays including DS (Minnes et al., 2015). Parents of children with DS who often used regenerative coping strategies, and who experienced positive personal changes in terms of posttraumatic growth suffered from less anxiety and somatization symptoms, whereas dysfunctional coping was the best predictor for parental depression and physical symptoms (Alexander & Walendzik, 2016).

Jameel and Malik (2022) found significant negative association of emotion-focused coping and children’s behavioral problem, while significant positive association of recently developed coping and children behavioral problem. Additionally, recently developed coping, family size, and mothers’ education were significant predictors of behavioral problems of the children with DS (Jameel & Malik, 2022). For mothers of children with DS, the children's levels of behavior problems, excitability and self-sufficiency were strongly related to outcome. Coping strategies, family relationships and socioeconomic factors also showed significant effects (Sloper et al., 1991).

## Study Strengths and Limitations

This is the first systematic review to our knowledge that has been conducted to examine religious coping among parents of children with DS in the literature. We used international guidelines to conduct this systematic review. This systematic review was very comprehensive in terms of search terms. It included English and non-English articles. The systematic review comprised large number of studies, large sample sizes, and participants from almost all major religions.

However, there were some limitations to this review. First, databases other than Pubmed and Scopus were not searched and dissertations/theses were also not included. Second, studies involving children with autism, developmental delay, and/or intellectual disability along with DS were also included. Third, although this study included an unbiased review of the major findings in the articles, it does not include a critical appraisal of each article. Lastly, the quality assessment was not performed because an investigator conducted the study selection process and review.

## Practical Implications and Recommendations

The current systematic review highlights the importance of religious coping for parents of children with DS, which is often overlooked in clinical practice, and will therefore draw the attention of health professionals to this issue. Knowing various coping strategies of parents will be effective in the care and management of children with DS. Raising a child with DS is a life-changing experience that spurs most parents to examine their belief systems.

Parents' religious beliefs become more important in times of crisis; therefore, the religious beliefs of parents of children with DS ought to be taken into account. Many parents of children with DS noted support from their religious community or place of worship; therefore, health professionals should not shy away from inquiring about other sources of support. Parents’ expressions of spirituality should be screened for and respected by health professionals. In other words, in addition to medical anamnesis, a spiritual anamnesis should be taken from parents of children with DS.

Parents should be informed about religious/spiritual support and encouraged to receive spiritual support. Parents should definitely be asked if they want any religious/spiritual support. When the spiritual needs are indicated, referral to a chaplain should be uncontroversial. A suitable environment should be prepared for parents to fulfill their religious duties and their requests for pastoral care should be implemented. On the other hand, most health professionals do not have sufficient knowledge about religion, spirituality, and religious coping. Therefore, the health professionals must be educated about religious coping, spiritual support and care with course modules. Furthermore, spirituality lessons should be taught in medical faculties, as in many universities in the USA.

Children's hospitals should have clergy, or preferably staff-appointed chaplains, who are experienced in caring for sick children and parenting. Parents whose children have been diagnosed with DS should be introduced to parents of children who have been diagnosed with DS in previous years. Religious and spiritual support seminars should be given to parent groups and their DS children in hospitals. Children's hospitals should have books with religious and spiritual content for DS children and their parents. Lastly, we strongly recommend that spiritual screening and signposting should be integrated within clinical genetic practices by administrators in the government and ministry of health.

## Conclusions and Future Research

Being a parent of a child with DS has many challenges; therefore, most parents of children with DS have high level of stress, anxiety and depression. The types of stressors change over time according to the child’s age, and coping strategies varied accordingly. Stress level of the parents is positively correlated with the number of important unmet needs. Great use of coping strategies is associated with less stress and fewer important unmet needs. Since religious practices have physical, mental, psychological and sociological benefits, they gain a positive perspective on life and provide individuals with resilience in coping with a stressful situation. It can also bring friendship, and emotional and practical support through religious communities and organizations.

Religious coping has been used by parents of children with DS in many cultures around the world, regardless of religion, race, or ethnicity. Mothers are more likely to turn to religion than fathers in coping strategies. Religion plays an important role in the lives of most parents of children with DS. Most parents of children with DS use religious beliefs to explain the reason for their child’s condition. Faith, religion, and spirituality are a source of support for many parents of children with DS. Belief in God, belief in fate and belief in the afterlife provide physical, mental and psychosocial relief for most parents of children with DS.

Religious practices often bring parents of children with DS a sense of hope, meaning, solace, strength, and control over their situations during difficult times. Religious coping reduces the frequency of depression and anxiety in parents of children with DS and increases quality of life and life satisfaction. Most parents effectively use coping strategies and adapt their lives to their new parental roles. Parents of children with DS frequently has a positive attitude. Most parents report that their child with DS has a positive impact on their and their family's lives and that they are happy to have the child.

Regarding future research, we believe that the following studies to be conducted will contribute greatly to the literature in the future: (1) stress factors and religious coping strategies in parents of children with DS of different ethnic origins and religions: A multicenter study; (2) comparison of psychosocial and mental functions of parents of children with DS who use and do not use religious coping; (3) use of religious coping in siblings of DS children and its effects on psychosocial and mental functions; (4) use of religious coping in grandparents of children with DS; (5) comparison of religious coping strategies in parents (and/or other family members) of children with DS before and after the spiritual support training program; (6) use of religious coping in older children with DS and its effects on physical, mental and psychosocial functions.

## Data Availability

Not applicable.

## Appendix 1

Studies About Religious Coping Among Parents/Caregivers of Children with Down Syndrome

**Table:**

Study no/Reference/Obtained from	Type of study	Sample size	Country of study/Ethnic background of participants	Religion of participants	Used stress, anxiety, depression, coping scales etc Significant stressors	Major findings of study
1/Herken et al. (2000)/Articles’ reference list	Questionnaire-based study	Forty-two parents (21 mothers and 21 fathers) of children with Down syndrome (DS) and control subjects	Türkiye/Turkish	Muslim	Beck depression inventory, perceived stress scale and coping orientation to problems experienced (COPE) were used No stressor was noted	The depression score of the parents of children with DS was found to be higher than the control group. The mothers had higher depression scores than fathers. Fatalism-religious behavior scores were higher, dependence, avoidance, social support, planning and active behavior scores were lower in the study group than the control group. Of the parents, 61.9% had sufficient knowledge about DS
2/King (2001)/ Articles’ reference list	Semi-structured interviewing questionnaire-based study	Thirty-four parents (32 mothers and two fathers) of adult children with disabilities, some with DS	USA/ African American	Religion of participants was not noted	The interview data were analyzed using the grounded theory process The stressors were personal, interpersonal (social), and structural levels in the lives of the parents	Many parents spoke of three strategies to cope with stressors: “a reliance on religious faith,” “a reliance on family support,” and “a positive assessment of even the most difficult situations.” Many thought they simply could not have survived the rigors of their caregiving careers without God’s help. Personal spirituality was a more effective coping strategy than reliance on church support
3/Lam and Mackenzie (2002)/Pubmed	Semi-structured qualitative interviews	Eighteen mothers of children with DS	China/ Chinese	Participants believed Confucian teachings	The Chinese parental coping scale was used: problem-focused coping (self-reliance, appeal to a supernatural power, seeking social support) and emotion-focused coping (avoidance) Major stressors were unexpected birth of an abnormal child, acceptance of the child, special needs of the child, worried about the future, knowledge deficit, marital relationship, and social restrictions	Coping strategies frequently used were avoidance, self-reliance, and seeking social support. The types of stressors changed over time according to the child’s age, and coping strategies varied accordingly
4/Sullivan (2002)/Pubmed	Questionnaire-based study	One hundred and fifty participants consisting of 78 males and 72 females, who were parents of children with DS	United Kingdom/ Ethnic origin of participants was not noted	Religion of participants was not noted	Carver, Scheier and Weintraub’s COPE inventory was used No stressor was noted	Mothers of children with DS recorded higher scores than fathers in the following areas of coping styles: “planning,” “seeking instrumental social support,” “seeking emotional social support,” “suppression of competing activities,” “turning to religion,” and “focus on and venting of emotions”
5/Van Riper (2007)/Pubmed	Questionnaire-based study	Seventy-six mothers who had a child with DS	USA/The majority (95%) of families were White	Religion of participants was not noted	The family-crisis-oriented personal evaluation scales was used The stressors with the highest percentage were as follows: increase in the amount of “outside activities” in which the children are involved; increase in the number of tasks or chores that do not get done; increased strain on family “money” for food, clothing, energy, and homecare; a family member purchased a car or other major items; and increase in arguments between parent(s) and children	Most mothers reported that their family was doing well or very well. Three family variables (i.e., family demands, family resources, and family problem-solving communication) were significantly associated with family adaptation. Of the five items from the family-crisis-oriented personal evaluation scales, the highest mean score was having faith in God
6/Fatima and Suhail (2010)/ Pubmed	Questionnaire-based study	One hundred mothers of children DS and 100 mothers of normal children	Pakistan/Pakistani	Muslim	Trait well-being inventory, mood level scale, hospital anxiety and depression scales, belief in a just world scale, scales of belief in immanent and ultimate justice were used No stressor was noted	Personal belief in a just world positively predicted life satisfaction and mood level and negatively predicted state anxiety and depression in both groups of mothers. In contrast, beliefs in immanent and ultimate justice were not consistently adaptive. The more the mothers of a DS child believed in immanent justice, the more anxiety they experienced the previous week. The more the mothers of normal children believed in ultimate justice, the more they experienced anxiety
7/Norizan and Shamsuddin (2010)/Pubmed	Self administered questionnaire-based study	One hundred and forty-seven mothers of children with DS	Malaysia/Malaysian	Religion of participants was not clearly noted. However, probably most of the participants were Muslim because majority of population is Muslim in Malaysia	Parental stress scale, pediatric symptom checklist, COPE inventory and DASS21 were used The stressors were children’s behavioral problems and maternal factors	Mean parenting stress among mothers of children with DS significantly differed by behavioral problems in their children. Parenting stress is significantly correlated with frequent use of acceptance, religious and optimist coping styles, and presence of maternal depressive, anxiety and stress symptoms. The most frequently used coping styles were religious coping, acceptance, optimist, and active coping
8/Pillay et al. (2012)/Pubmed	An observational qualitative study, based on in-depth interviews	Eight mothers of children with DS	Australia/Australian	Religion of participants was not noted	The interview guide, using interviews in a research project was used Significant stressors were children’s behavioral problems, health conditions, society’s misconceptions, finding a work-life balance, financial stress, schooling and what does the future hold	Overall spirituality was described as a stronger and more dynamic source of support than organized religion in coping with stressors and life’s challenges associated with raising a child with DS. However, both were useful in mediating stress and supporting mental health particularly during stressful life events. Data analysis revealed that spirituality and religion for women could be best conceptualized as a source of support
9/Sheets et al. (2012)/Pubmed	Semi-structured qualitative interviews	Fourteen mothers of children with DS	USA/Latinas	Mothers who disclosed their religious orientation attended Catholic or Latter-Day Saints churches	Breaking difficult news was used: religious coping, various supports, and engendered feelings The major stressors were lack of knowledge and lack of support	Six significant themes emerged: “cultural belief system,” “communication,” “support/ lack of support,” “feelings engendered,” “medical issues,” and “medical system.” Religiosity was the most prominent theme from the mothers. They used religious beliefs to explain the reason for their child’s condition. Most mothers came to believe that their child is a blessing. Intertwining religiosity with other beliefs about DS seemed to play a role in bonding with their child
10/Gupta et al. (2013)/Pubmed	Questionnaire-based study	Twenty-five parents (14 mothers and nine fathers) of children with DS	India/Indian	Religion of participants was not noted	Cognitive emotion regulation questionnaire was used No stressor was noted	It appeared that both mothers and fathers have good acceptance for the situation and have used refocus on planning (cognitive part of action-focused coping), rumination, catastrophizing, positive reappraisal and putting into perspective strategies almost equally. Probably, religious and spiritual beliefs also help in coping strategies of parents of children with DS
11/Nelson Goff et al. (2013)/ Pubmed	Web-based survey (questionnaire)	One hundred and fifteen parents (103 females and 12 males) of children with DS diagnosed postnatal	USA/Most participants were European American	Religion of participants was not clearly noted; however, most probably most of the participants were Christian	The survey included both quantitative measures and qualitative questions Major stressors were lack of information provided by doctor and lack of support	Faith played a large role in many of respondents’ process of adjustment; for some couples it was their “faith in God that He would give us the grace to deal with the medical, physical, cognitive and social challenges-and He has!” Some parents stated that their personal faith was a large part of their adjustment and describing their child as a blessing or gift from a higher power
12/Gallagher et al. (2015)/Articles’ reference list	Questionnaire-based study	Thirty-two parents (females and males) of children with developmental disabilities, seven with DS	United Kingdom/Caucasian	In qualitative study, five parents from the main sample identified themselves as Roman Catholics (2), Christian (1), Muslim (1), and mixed religious heritage including Muslim and Christian (1), similar reflection of the quantitative sample	The hospital anxiety and depression scale, the beliefs and values scale, and support functions scale were used No stressor was noted	Spiritual/religious coping was more likely to be used as a means of seeking escape from their caregiving demands and as a way of dealing with stress. Spirituality proved to be a mediator of the social support and depression association; parents who reported less social support had greater spiritual beliefs. However, parents who held stronger spiritual beliefs reported more depressive symptoms
13/Gören (2015)/ Articles’ reference list	Semi-structured qualitative interviews	Thirteen mothers of children with DS	Türkiye/Turkish	Muslim	Interviews were carried out Major stressors (needs) were related to child caregiving burdens, familial difficulties, and challenges related to social, spiritual, and professional support	Internal sources that support mothers' needs were making sense of the situation and being able to change. External sources were support from spouse, other family members, friends, neighbors and institutional supports. Religious coping styles used by the mothers were finding meaning, getting social support, focusing on postponement (patience), believing in divine justice, and practicing submission/cooperation. All mothers stated that they gained one or more meanings related to their children's presence in their lives
14/Hayat and Zafar (2015)/ Articles’ reference list	Questionnaire-based study	One hundred and twenty parents (60 mothers and 60 fathers) of children with DS	Pakistan/Pakistani	Muslim	Hospital anxiety depression scale, parental stress scale, and brief COPE were used No stressor was noted	The most used coping strategy in parents with DS children was active avoidance coping. Results showed significant correlations between psychological well-being and coping strategies. Those parents who relied more on active avoidance coping, reported lower levels of psychological well-being as compared to those who relied on problem-focused coping strategies. Fathers scored significantly high on psychological well-being than mothers. Religious/denial coping was negatively associated with depression but no significant correlation was found between religious/denial coping and anxiety and stress
15/Alcantara and Castronuevo (2016)/Articles’ reference list	Semi-structured qualitative interviews	Five families having a child with DS: Ten participants, two members from each family (one parent and one sibling)	Philippines/Filipino	Christian	Qualitative design was used Main stressors were financial and health problems	The struggles were categorized into five issues: “lifestyles adjustment,” “struggles with relationships,” “financial problems”, “responsibilities,” and “health conditions.” The coping of the families were psychological, physical, psychosocial, and psychospiritual. All of the respondents shared that they always pray to God and that their families go to church in order to keep their hearts and wills strong and resilient. They also mentioned God as one of the motivations they have to keep on moving forward
16/Behrani and Shah (2016)/ Articles’ reference list	Questionnaire-based study	Thirty parents (15 mothers and 15 fathers) of children with autism/ intellectual disability/DS	India/Indian	Religion of participants was not noted	COPE questionnaire and an informal interview schedule were used Major stressors were having a special child and children’s behavioral difficulties	No significant difference was found between mothers and fathers of children with disability for coping patterns. However, positive reinterpretation and growth coping pattern were used more frequently by fathers and behavioral disengagement coping pattern were used more frequently by mothers of children with disability. Mothers were turning more toward to religious coping, whereas fathers were adopting more accepting coping pattern
17/Nelson Goff et al. (2016)/Scopus	Web-based survey (questionnaire)	Four hundred and forty-five participants (239 males and 206 females) of children with DS	USA/non-Hispanic White (90.7%), non-Hispanic Black (1.0%), Hispanic (4.3%), non-Hispanic other (3.8%)	Religion of participants was not clearly noted; however, probably most of the participants were Christian	Family crisis oriented personal evaluation scales, Herth hope index, satisfaction with life scale, revised dyadic adjustment scale, and the couples satisfaction index were used The stressors were child’s developmental level, acceptance of the diagnosis, society’s view of their child, fear of the future, and resources	Key areas that parents reported were acceptance of the diagnosis, having a positive attitude, their child’s developmental level, and other internal and external factors that contribute to their attitudes and coping. When coping with their child’s diagnosis, some parents of children with DS frequently citied their religion or being spiritual as important. Some participants described a stronger faith brought about from their spiritual growth
18/Barros et al. (2017)/Pubmed	Questionnaire-based study	One hundred and sixty-eight caregivers of 84 children with DS and 84 children without disability. Most caregivers were mothers	Brazil/Brazilian	Catholic (43%), Evangelical (31%), Spiritist (5%), and no religion (21%)	Zarit burden interview scale was used. It evaluates physical and emotional health, psychological well-being, social life, and financial situation of the caregiver Major stressors were fear about the future, dependence of child/adolescent, and financial difficulties	The caregivers of children with DS presented a moderate burden compared to the caregivers of normoreactive children. Religious belief has a fundamental effect and acts by modifying the world view of the individual, using it as a driving force for better coping with and better overcoming everyday difficulties, minimizing the burden, anguish, and stress of the caring process
19/Huiracocha et al. (2017)/Pubmed	In-depth qualitative interview	Eight parents (mothers and fathers) of children with DS	Ecuador/Ecuadorian	Catholic	In-depth interviews were carried out Major stressors were lack of social support and widespread stigmatization	Faith and religious observance are central for the parents. Religious beliefs have a complicated significance. For some mothers, faith served as an important resource in making sense of their child’s condition. On the one hand, religious beliefs render mothers vulnerable to the chastisements of priests
20/Isa et al. (2017)/Scopus	Self-administered questionnaire-based study	One hundred and ninety caregivers (father, mother grandfather, grandmother, siblings or others) of children with learning disabilities, 100 with DS	Malaysia/Malaysian	Religion of participants was not noted; however, probably most of the participants were Muslim, because most Malays are Muslims	The perceived stress scale and the brief COPE scale were used No stressor was noted	Caregivers of children with learning disabilities had slightly higher levels of stress than average recommended. The most frequently employed coping styles among caregivers were religion, acceptance, and positive reframing. Higher perceived stress was significantly predicted by frequent use of instrumental support and behavioral disengagement coping styles. Caregivers with more children and those with frequent use of emotional support and religion coping were found to have lower perceived stress
21/Cless et al. (2018)/Pubmed	Web-based survey (questionnaire)	Three hundred and fifty-one mothers of children with DS	USA/ European American: White (90.3%), Latino/Hispanic (4.3%), African American (0.6%), American Indian or Alaska Native (0.9%), Asian or Pacific Islander (0.6%), other (2.8%)	Protestant (41.9%), Catholic (24.5%), Jewish (4.3%), nondenominational (15.7%), none (9.4%) and other (3.1%)	Family crisis oriented personal evaluation scales, Herth hope index, the revised dyadic adjustment scale, and the couples satisfaction index were used No stressor was noted	A greater degree of religious coping and internal coping were each significantly associated with more hope, whereas support seeking was not related with more hope. Higher hope was significantly associated with greater relationship quality. Bootstrapped indirect effects from both religious coping and internal coping to hope, and then hope to relationship quality, were identified
22/Amireh (2019)/Scopus	Questionnaire-based study	One hundred and seventy-one parents (mothers and fathers) of children (88 with typically developed, 55 with autism and 28 with DS)	Jordan (Asia)/Jordanian	Muslim	Parenting stress index–short form, and brief COPE were used Major stressors were parental distress, parent–child dysfunctional interactions and difficult child	Practicing religion was the most widely used coping strategy by the parents of DS children. Parents’ age, location, and income; child’s age and gender; and parents’ relationship with child had no effect on the total level of stress experienced by parents
23/Barakat and Mohamed (2019)/Articles’ reference list	Structured interviewing questionnaire-based study	Fifty parents (females and males) of children with DS	Egypt/Egyptian	Muslim	Structured interviewing questionnaire sheet, parental stress scale, brief-COPE inventory, and Ryff’s psychological well-being scale were used Main stressor was financial problems	Nearly to half of the parents had high level of stress, while more than one third of them had moderate level of stress. More than half of the parents had low level of their coping strategies, two thirds had low level of psychological wellbeing. There was a correlation between religious coping and total of psychological wellbeing among parents with DS children. There was also a correlation between religious coping and purpose in life and self-acceptance
24/Boehm and Carter (2019)/ Articles’ reference list	Questionnaire-based study	Five hundred and thirty parents or primary caregivers of individuals with intellectual disability, 100 with DS	USA/Mostly white (non-Hispanic) (86.5%) The others were African American/ Black, Latina/ Latino/Hispanic, Asian/Asian American, American Indian or Alaska Native, and others (e.g., Middle Eastern)	Most participants (90.8%) identified with a particular religion. The most common were Catholic/Roman Catholic, Baptist, non-denominational Christian. Others were Methodist, Lutheran, Presbyterian, Church of Christ, Bible Church, Episcopal, Jewish, and Pentecostal	Children’s needs were assessed using a measure of support needs. Religious/ spiritual beliefs, practices, and social supports were assessed using the systems of belief inventory. Strength of religious faith was assessed using the Santa Clara strength of religious faith questionnaire – short form No stressor was noted	Religion and spirituality held a prominent place in the lives of most of the parents and caregivers. In the sample, 92% affirmed the existence of God in some form, most said they prayed or meditated, and their strength of religious faith was found to be moderately strong. Of participants, 84% indicated these same practices brought them peace of mind, 83% of participants said they experienced a sense of hope as a result of their beliefs, 74% participants agreed that “I believe God will not give me a burden I cannot carry” and 73% reported that prayer or meditation helped them cope in times of stress
25/Qahhar and Khan (2019)/ Articles’ reference list	Self-administered questionnaire-based study	Two hundred parents (100 mothers and 100 fathers) of children with DS	Pakistan/Pakistani	Muslim	Depression, anxiety and stress scales and brief religious coping scale were used: stress, depression, anxiety, positive religious coping, and negative religious coping No stressor was noted	Religious coping helps parents in minimizing their psychological distress. The strong positive connection with Allah (Subhanahu Wa Taala) brings in comfort and hope needed to take care of the needs of their child. This study confirmed the fact that inward harmony can be preserve through the religious belief system and religious mode of coping mechanism in parents, dealing with major life stressor
26/Gashmard et al. (2020)/Pubmed	Semi-structured interviews	Twenty family members (ten mothers, six fathers, two brothers, and two sisters) of children with DS	Iran/Iranian	Muslim	Semi-structured interviews were used Stressors were related to child caregiving burdens, familial difficulties, and challenges related to social and professional support	Six categories were extracted: “searching for information,” “paying attention to children's healthcare needs,” “concentration on spirituality,” “teaching socially appropriate behavioral skills,” “efforts to increase self-reliance in children,” and “development of family support circle.” The participants believed that their spiritual and religious beliefs played a fundamental role in adaptation with the conditions of their children with DS
27/Mohammed et al. (2020)/ Articles’ reference list	Questionnaire-based study	One hundred and twenty family caregivers (110 mothers and 10 fathers) of children with DS	Egypt/Egyptian	Muslim	Interviewing questionnaire, Ryff’s psychological well-being scale and brief COPE inventory were used Stressors were financial difficulties, stigmatization of child and lack of socialization	The most frequent used emotion-focused coping strategies were acceptance and religion through accepting the reality and becoming satisfied of it, considering the child disability as a test from Allah, seeking help from Allah to solve the child problems. It may be attributed to good faith and religiosity that are highly prevalent in the community where a disabled child might be considered as a gift from Allah. There was a negative significant correlation between level of psychological well-being and emotion-focused coping strategies except for use of emotional support, acceptance, self-distraction, venting, humor, and religion
28/Suza et al. (2020)/Articles’ reference list	An observational qualitative study, based on in-depth interviews	Ten mothers of children with DS	Indonesia/ Bataknese (4) Javanese (4) Malaynese (2)	Muslim (9), Christian (1)	In-depth interviews were performed The data was evaluated using the Collaizi content analysis method Stressors were related to child caregiving burdens and worries about the future	Five themes were recognized as follows: “having an emotional experience,” “accepting the child’s situation,” “meeting the needs of children,” “experiencing obstacles in caring for children,” and “expectation of the mother.” Participants tend to accept the condition with patience and sincerity, despite the initial refusal at first, on learning about the child’s DS. Some participants had a feeling of persistent gratitude and fortune toward Allah in providing care for the child with DS
29/Braga et al. (2021)/Pubmed	A semi-structured interview script based on the adopted theoretical framework	Thirty-nine mothers and three fathers of children with DS	Brazil/Brazilian	Religion of participants was not noted	It is a qualitative research guided by the resiliency model of family stress, adjustment, and adaptation The stressors were related to child caregiving burdens, financial difficulties, familial difficulties, worries about the future, challenges related to social and professional support	Problem-solving and coping proved to be a set of actions, behaviors, efforts, and communications that contributed to family adaptation and favored balance between the demands imposed by DS and the acquisition of resources by the family. As external resources that represent the search for assistance or support, the authors found a search for information, initially about DS and over time about stimulation and inclusion, search for health care, rights and support groups, adoption of strategies for stimulation of the child, as well as belief in God
30/Darla and Bhat (2021)/Pubmed	Questionnaire-based study	Fifty-one parents (mothers and fathers) or caregivers of DS children	India/Indian	Hindu (82.3%), Muslim (13.7%), Christian (1.9%), Buddhist (1.9%)	The PedsQL family impact module questionnaire and COPE inventory questionnaire were used The stressors were worry and conditions related to communication, social functioning, daily activities, and family relationships	The mean score of the quality of life of the families was 68%. The least and the most affected domains were cognitive functioning and worry, respectively. Religious coping, acceptance and emotional social support were foremost coping strategies. Though 27% were upset with the diagnosis, most had a “feeling of love” toward the child (72%). Of participants, 50% had limited knowledge about DS and lacked organizational support (60%)
31/Ezeonu et al. (2021)/ Scopus	Cross-sectional interviewing questionnaire-based study	Forty caregivers (28 females 12 males) of children with a disability, six with DS	Nigeria/Nigerian	All were of Christianity religion with half of them of catholic denomination (50%), 10% Anglican while the rest were distributed across several other denominations	The standard COPE inventory developed by Carver et al was used The major stressors were financial difficulties and limited social supports	Turning to religion, planning, acceptance, positive reinterpretation, active coping, and seeking social support were the commonest coping strategies used by the caregivers. Turning to God and hoping for the best was significantly related to the forms of disabilities. The findings support that religious belief provides endurance and resistance to people dealing with stress
32/Karaca and Konuk Şener (2021)/Scopus	In-depth qualitative interviews	Twenty-eight mothers of children with developmental disabilities, some with DS	Türkiye/Turkish	Muslim	A semi-structured form created by the researchers to guide and lead the interviews was used The most important stressor was concerns about the future	The study revealed four main themes: “the journey to acceptance,” “the meaning/ purpose of life,” “concerns about the future,” and “coping strategies.” Religious coping included relying on Allah, dua, salah (namaz), and reading the Quran. By virtue of spirituality, the mothers were able to survive the troublesome days and cope with the stress of their new lives. They began to see their children as giving meaning to their lives, experienced enhanced feelings of love for their children, and commitment to each other
33/Lee et al. (2021)/Pubmed	Questionnaire-based study	One hundred and fifty-two parents/ caregivers (133 mothers, 16 fathers and three caregivers) of people with DS	Canada/ White (87% of participants), South Asian, Hispanic/Latin, American, Aboriginal, Black, Multi-racial, Southeast Asian, other	Religion of participants was not noted. Probably most of the participants were Christian, because the subscale of seeking spiritual support included an example of attending church services	Demographics questionnaire, family needs survey, questionnaire on resources and stress-Friedrich version, and family crisis oriented personal scales were used Important stressors (unmet family needs) were family and social support, explaining to others, child care, financial, information, community services, and professional support	Stress level was positively correlated with the number of important unmet needs, and great use of coping strategies were associated with less stress and fewer important unmet needs. Reframing and passive appraisal strategies had significantly higher scores than mobilizing family support, acquiring social support and seeking spiritual support. Mobilizing family support was rated significantly higher than acquiring social support and seeking spiritual support
34/Mohammed et al. (2021)/ Articles’ reference list	Questionnaire-based study	Sixty mothers of children with DS	Egypt/Egyptian	Muslim	Interviewing questionnaire, brief-COPE inventory, and Ryff’s psychological well-being scale were used The major stressor was financial difficulties	The most frequent used emotion-focused coping strategies were religion, acceptance and venting while the most frequent used problem-focused coping strategy was use of instrumental support. More than half of mothers of children with DS had psychological well-being less than usual. There was a negative statistically significant correlation between psychological well-being and all types of emotion-focused coping strategies except for humor, acceptance and religion
35/Pruktarat et al. (2021)/Scopus	Interviewing questionnaire-based study	One hundred and twenty mothers of children with DS	Thailand/Thai	Of participants, 95% were Buddhist	The demographic interviewing questionnaire, family stressors and strains questionnaire, family hardiness index, social support questionnaire, family crisis oriented personal evaluation scales, and Chulalongkorn family inventory were used The stressors were increased difficulty in providing care and in managing of children	Most mothers got their support from family members and the most common type was emotional support, followed by tangible support. Reframing (or redefining stressful events to make them manageable) was the most used strategy for family problem-solving and coping. The others, in order of frequency, were acquiring social support, seeking spiritual support, mobilizing family support, and passive appraisal
36/Al Rubaie (2022)/Pubmed	Questionnaire-based study	Twenty mothers of children with DS	Saudi Arabia/Saudi	Muslim	A questionnaire was set up after reviewing the literature on early intervention programs and children with DS Stressors related to social, psychological, educational, and cognitive qualifications were noted	Cognitive qualifications were highest at an average of 52% and religious qualification at an average of 48%. The most prominent phrase in religious qualification was strengthening mothers’ belief that their children are the key to reinforcing mothers’ will to accept Allah’s will and destiny
37/Duarte et al. (2022)/Pubmed	An observational qualitative study, based on in-depth interviews	Forty-two parents (39 mothers and three fathers) of children with DS	Brazil/Brazilian	Religion of participants was not noted. However, it was noted that in Brazil, the main religions are Roman Catholic, Evangelical, Spiritualist, and Jehovah’s Witness, respectively	The consolidated criteria for reporting qualitative research guideline was used Major stressor was child caregiving burdens	Seven themes were founded: “meaning and purpose in life of children with DS,” “hope and family strength,” “trust and connection,” “spiritual practices,” “the concept of God or other higher Being (Divinity),” “personal beliefs and values,” “meeting the needs of love,” and “contribution to the transformation in life.” Spirituality was a resource in parents of children with DS. Participants referred to God as support and transformation, always providing improvements in the lives of families, and providing the necessary resources to face their difficulties. They consider that there is a purpose of God for them to be exposed to this situation
38/Elmwafie et al. (2022)/Articles’ reference list	A quasi-experimental (one group pre-test/post-test) design	Seventy-five mothers and their children with DS	Egypt/Egyptian	Muslim	A structured interviewing questionnaire, self-reliance assessment, needs’ scale, coping patterns scale and family quality of life scale were used The major stressor was financial difficulties	Religious coping was the most used strategy at pre-intervention and post-intervention programs. A highly statistically significant improvement was observed between pre-intervention and post-intervention programs for mothers’ knowledge, quality of life, needs, and coping patterns. Implementation of coping strategies intervention had a positive effect on the improvement of mothers’ knowledge, level of needs, coping patterns and quality of life
39/Gokgoz and Kabukcuoglu/(2022)/Scopus	Semi-structured in-depth interviews	Fifteen mothers of children with DS	Türkiye/Turkish	Muslim	The classic grounded theory approach was employed The stressors were child caregiving burdens, information deficits, lack of social and professional support	Three categories emerged: “reconstruction of mothering,” “factors affecting the process,” and “response to the changing life.” The most obvious coping methods used by mothers were religiosity and peers who are experiencing similar situations. The mothers explained that they experienced personal growth, such as feeling stronger, improving their ability to communicate, and becoming “closer to Allah”. The mothers said their belief in Allah had increased. They explained that Allah, prayer and the Quran supported them in this process
40/Mahmoud et al. (2022)/ Articles’ reference list	Structured interviewing questionnaire-based study	Ninety-five primary students with DS and their mothers	Egypt/Egyptian	Muslim	Structured interviewing questionnaire, physical examination sheet mothers' coping scale and mothers' needs scale were used The major stressor was financial difficulties	Religious and doctrinal coping was the most coping strategy used by mothers, in which more than half of mothers were always able to cope religiously. Three fifths of mothers had negative coping patterns, in which more than two fifths of them never able to cope with their children disability. More than two-thirds of mothers had unsatisfactory level of knowledge regarding of DS
41/Negm et al. (2022)/Articles’ reference list	Structured interviewing questionnaire-based study	One hundred mothers of children with DS	Egypt/Egyptian	Muslim	Structured interviewing questionnaire, Ryff’s psychological well-being scale, mothers' needs scale, and mothers' coping scale were used The major stressor was financial difficulties	Religion is widely acknowledged as a major support system for moms in this population when coping with a stressful situation. Nearly two-thirds of mothers of children with DS had psychological well-being less than usual pre-educational intervention compared to more than half of mothers of children with DS had psychological well-being better than usual post-educational intervention. Three-fifths of mothers had negative coping patterns, with more than three-quarters of them never being able to manage their children's disabilities
42/Özkan and Günaydın (2022)/Articles’ reference list	An observational qualitative study, based on in-depth interviews	One hundred and six families (mothers and fathers) of with disabled children, 29 with DS	Türkiye/Turkish	Muslim	In-depth interviews, personal information form, Ok-religious attitude scale and Diener-satisfaction with life scale were used Stressors were related to child caregiving burdens, familial difficulties, and worries about the future	Five themes were recognized: “religious beliefs,” “causes of acceptance”, “reasons for being negative,” “coping with problems,” and “family acceptance and support.” Religious beliefs included believing that it is a fate, seeing the care as a good deed, seeing the care as a test, and not having any other choice but believing. Religious attitude positively affects people’s life satisfaction
43/Celik and Kara Uzun (2023)/Pubmed	Semi-structured interviewing questionnaire-based study	Twenty-six parents (23 mothers and three fathers) of children with DS	Türkiye/Turkish	Muslim	Semi-structured interviews were conducted to explore parents' stressful experiences, possible adaptations, and coping strategies to overcome the challenges Six stressful themes were caregiving burdens, struggle against stigma and discrimination, concerns about the future, challenges related to health and education services, and financial difficulties	Religious and spiritual factors played an essential role in helping early acceptance. Almost all parents reported that their religious beliefs encouraged them to feel better, be strong, and accept the diagnosis. They believed that their child was given by the decree of Allah and that having a child with DS was divine providence. Some parents stated that they saw their child as happiness, beauty, a gift, trust, fate, and an opportunity/trial to gain paradise
44/Hafez et al. (2023)/Articles’ reference list	Structured interviewing questionnaire-based study	One hundred mothers of children with DS	Egypt/Egyptian	Muslim	Structured interviewing questionnaire, mothers' needs and mother coping patterns were used. The coping patterns of mothers were physical coping, psychological coping, social coping, emotional coping, educational coping, and religious and doctrinal coping including pray and put my trust in Allah, ask Allah for healing by practicing religious rites, etc. The stressors were cognitive, economical, physical, psychological and social needs	Three fourths of mothers had moderate economical needs and more than half of mothers had very much physical, psychological and social needs. More than one third of mothers had positive coping pattern with their children suffering from DS and near two thirds of mothers had negative coping pattern with their DS children. There was a positive correlation between total knowledge score and tot al coping pattern
